# Ribosomal protein L10(L12)_4_ autoregulates expression of the *Bacillus subtilis rplJL* operon by a transcription attenuation mechanism

**DOI:** 10.1093/nar/gkv628

**Published:** 2015-06-22

**Authors:** Helen Yakhnin, Alexander V. Yakhnin, Paul Babitzke

**Affiliations:** Department of Biochemistry and Molecular Biology, Center for RNA Molecular Biology, The Pennsylvania State University, University Park, PA 16802, USA

## Abstract

Ribosomal protein genes are often controlled by autoregulatory mechanisms in which a protein encoded in the operon can either bind to newly synthesized rRNA during rapid growth or to a similar target in its mRNA during poor growth conditions. The *rplJL* operon encodes the ribosomal L10(L12)_4_ complex. In *Escherichia coli* L10(L12)_4_ represses its translation by binding to the *rplJL* leader transcript. We identified three RNA structures in the *Bacillus subtilis rplJL* leader transcript that function as an anti-antiterminator, antiterminator or intrinsic terminator. Expression studies with transcriptional and translational fusions indicated that L10(L12)_4_ represses *rplJL* expression at the transcriptional level. RNA binding studies demonstrated that L10(L12)_4_ stabilizes the anti-antiterminator structure, while *in vitro* transcription results indicated that L10(L12)_4_ promotes termination. Disruption of anti-antiterminator, antiterminator or terminator function by competitor oligonucleotides *in vitro* and by mutations *in vivo* demonstrated that each structure functions as predicted. Thus, *rplJL* expression is regulated by an autogenous transcription attenuation mechanism in which L10(L12)_4_ binding to the anti-antiterminator structure promotes termination. We also found that translation of a leader peptide increases *rplJL* expression, presumably by inhibiting Rho-dependent termination. Thus, the *rplJL* operon of *B. subtilis* is regulated by transcription attenuation and antitermination mechanisms.

## INTRODUCTION

Bacterial survival depends upon the capacity to rapidly and profoundly alter metabolism, physiology and behavior in response to changing environmental conditions. These responses involve regulated adjustments in the expression of numerous genes. Transcription is regulated at the level of initiation, elongation and termination. Two common transcription termination mechanisms have been described. In Rho-dependent termination, Rho triggers transcript release when it encounters paused RNA polymerase (RNAP), while intrinsic termination can occur in the absence of an accessory factor (1). Canonical intrinsic terminators consist of an uninterrupted RNA hairpin followed by a U-rich tract, with transcript release occurring 7–9 nucleotides downstream from the hairpin (2). A variety of transcription elongation factors, including NusA and NusG, regulate transcription by affecting RNAP pausing and termination (1-5). NusG enhances Rho-dependent termination by serving as a bridge between Rho and RNA polymerase (6), while NusA increases the intrinsic termination efficiency by reducing the transcription rate and by stimulating hairpin folding (2,7). It was recently shown that NusG is also capable of stimulating intrinsic termination, although this has only been observed for mycobacterial RNAP (8).

Transcription attenuation controls gene expression by regulating termination in the leader region preceding the structural gene(s). In attenuation, the action of a regulatory molecule induces transcription termination, with the default being transcription readthrough (9). A critical feature of several attenuation mechanisms is overlapping antiterminator (AT) and terminator (T) structures that form in the nascent transcript. As the AT precedes the T, formation of the AT prevents termination. Thus, interfering with AT formation by the regulatory molecule promotes transcription termination (9–12).

In addition to transcription attenuation, many bacterial genes are regulated post-transcriptionally by controlling translation initiation. Translational control mechanisms have been identified in which the action of a regulatory molecule promotes formation of an RNA hairpin that sequesters the Shine–Dalgarno (SD) sequence such that 30S ribosomal subunits are unable to interact with mRNA. In other instances, the regulatory molecule directly blocks ribosome binding because its binding site(s) overlaps the ribosome binding site (13).

Several ribosomal protein genes in *Escherichia coli* are regulated by autoregulatory feedback mechanisms in which one gene in the operon encodes a ribosomal protein that can either bind to rRNA during ribosome biogenesis or to its mRNA. When bound to its mRNA it can repress expression via transcription attenuation, or more commonly by repressing translation initiation (13–15). In these feedback mechanisms, the protein binding targets in rRNA and mRNA are similar in structure and constitute examples of molecular mimicry. During rapid growth the ribosomal protein binds to newly synthesized rRNA, whereas during poor growth conditions rRNA synthesis is reduced such that the free ribosomal protein instead binds to its mRNA target.

Although transcription attenuation and translation repression mechanisms autogenously control more than half of the ribosomal protein genes in *E. coli* (15), little is known about the mechanisms controlling ribosomal protein genes in Gram-positive organisms. In *Bacillus subtilis* only one such mechanism has been firmly established. In this case, ribosomal protein L20, encoded by *rplT*, binds to the untranslated leader of the *infC-rpmI-rplT* transcript and represses expression of the operon by a transcription attenuation mechanism (16). In addition to this attenuation mechanism, it appears that ribosomal protein S15, encoded by *rpsO*, represses its translation (17), while an S6-S18 complex, encoded by *rpsF* and *rpsR* respectively, appears to repress translation of the *rpsF-ssbA-rpsR* operon (18). Finally, ribosomal protein S4 (*rpsD*) binds to its untranslated leader transcript and represses its translation, although the precise mechanism is unknown (19,20).

The *rplJL* operon encodes ribosomal proteins L10 and L12 in a variety of bacterial species (21). These proteins form a complex containing one L10 subunit and four subunits of L12, which binds to 23S rRNA during ribosome biogenesis. In *E. coli*, this complex represses its own translation by binding to its mRNA upstream of the *rplJ* protein coding sequence (22,23). Although the mechanism of repression was not firmly established, it was proposed that L10(L12)_4_ binding alters the structure of the leader such that the SD sequence becomes sequestered in an RNA hairpin (23). More recent *in vitro* binding studies with *Geobacillus stearothermophilus* L10(L12)_4_ demonstrated that this protein complex interacts with common structural features found in 23S rRNA and the *rplJL* leader transcript, including a kink-turn motif and an unpaired UAA sequence within a secondary structure (21). Although it was proposed that *rplJL* operons from a variety of organisms including *G. stearothermophilus* and *B. subtilis* are regulated by a translational repression mechanism, the regulatory mechanism was not explored (21). We examined the regulatory mechanism of the *B. subtilis rplJL* operon and found that L10(L12)_4_ autoregulates its expression by transcription attenuation.

## MATERIALS AND METHODS

### Bacterial strains and plasmids

All *B. subtilis* strains used in this study are listed in Supplementary Table S1. The prototrophic *B. subtilis* strain PLBS338 has been described previously (24). Plasmids pTZ19R (Thermo Scientific), pQE80L (Quiagen), pDH32 (25), pTrpBGI-PLK (26) have been described. Plasmids pYH134 and pYH223 were constructed by cloning chromosomally derived polymerase chain reaction (PCR) fragments containing −382 to +200 or −382 to +231 relative to the start of *rplJL* transcription into the *Eco*RI and *Bam*HI sites of pTZ19R, respectively. Similarly, pYH244 was constructed by cloning positions −382 to +170 into pTZ18U. Plasmid pCA1 (*rplJ-lacZ* transcriptional fusion) was constructed by subcloning the *Eco*RI-*Bam*HI fragment from pYH134 into pDH32. Similarly, plasmids pCA2 (*rplJ’-’lacZ* translational fusion) and pYH221 (*rplLP’-’lacZ* translational fusion) were generated by subcloning the *Eco*RI-*Hin*dIII fragments from pYH223 or pYH244 into pTrpBGI-PLK, respectively. After linearization of pCA1, pCA2 and pYH221 with *Sca*I, each fusion was integrated into the chromosomal *amyE* locus of PLBS338 to generate strains PLBS655, PLBS656 and PLBS768, respectively.

The *B. subtilis-E. coli* shuttle vector pAY132 (pVector) contains the pAMα1 origin for replication in *B. subtilis*, the pUC origin for replication in *E. coli*, the tetracycline resistance gene from pHY300-PLK, a P_T7A1_-*lacO* IPTG-inducible promoter, as well as *lacI^q^* and the transcription terminators T_0_ and T_1_ from pQE80L. Plasmid pYH212 (pLeader) was constructed by cloning a PCR fragment containing the *rplJL* leader (−2 to +200 relative to the start of transcription) into the *Eco*RI and *Bsp*EI sites of pAY132 such that the IPTG-inducible promoter drives transcription. pYH213 (pL10–L12) contains a chromosomally derived PCR fragment containing the *rplJL* coding regions cloned into the *Xho*I and *Bsp*EI sites of pAY132 such that the IPTG-inducible promoter drives transcription of *rplJL*. Plasmid pYH216 (pLeader + L10–L12) was subsequently generated by subcloning the *rplJL* leader fragment from pYH212 into the *Bsp*EI and *Hin*dIII sites of pYH213.

Plasmid pYH217 was generated by changing the ATG start codon of the *rplJL* leader peptide (LP) to a TAG stop codon using the QuickChange mutagenesis protocol and pYH223 as template. Similarly, plasmids pYH231, pYH245 and pYH242 were constructed by inserting TTTGCCTC between C117 and C118, inserting TTGAGGTGTA between A72 and T73, or by replacing G131 and G132 with C residues, respectively, whereas plasmid pYH249 was generated by changing the LP translation initiation codon (ATG) in pYH244 to a stop codon (TAG). Lastly, plasmid pYH234 was generated by replacing the *B. subtilis* LP coding sequence in pYH223 with that of *Bacillus amyloliquefaciens* (Supplementary Figure S1). Plasmid pYH251 was then generated by changing the LP ATG start codon in pYH234 to a TGA stop codon.

Plasmids pYH219, pYH237, pYH238, pYH243, pYH246, pYH252 and pYH254 were constructed by subcloning *Eco*RI-*Hin*dIII fragments from pYH217, pYH234, pYH231, pYH242, pYH245, pYH249 and pYH251 into ptrpBGI-PLK. After linearization with *Sca*I, these fusions were integrated into the chromosomal *amyE* locus of PLBS338 to generate strains PLBS770, PLBS775, PLBS777, PLBS778, PLBS780, PLBS786 and PLBS788, respectively.

### L10(L12)_4_ protein purification

Plasmid pYH146 for 6His-L10(L12)_4_ overexpression was constructed by cloning a chromosomally derived PCR fragment containing the *rplJL* coding regions into the *Bam*HI and *Pst*I sites of pQE80L. *E. coli* strain JM83 was transformed with plasmid pYH146 containing *B. subtilis rplJL*, encoding N-terminal His-tagged L10 and native L12. Cells were grown in LB supplemented with 100 μg/ml ampicillin at 30°C and *rplJL* expression was induced with IPTG and grown for another 5 h. Cells were harvested and cell pellets were suspended in lysis buffer (100 mM Tris-HCl, pH 8.0 and 400 mM NaCl) and then lysed by sonication. Following centrifugation of the lysate at 20 000 g for 30 min, the supernatant was loaded onto a DEAE 52 column to remove cellular nucleic acids. The flow through was diluted 2-fold with water and mixed with Ni-NTA (Quiagen) that had been equilibrated with 50 mM Tris-HCl, pH 8.0 and 200 mM NaCl. Proteins were eluted with an imidazole step gradient, with the L10(L12)_4_ complex eluting in the 250 mM imidazole fraction as determined by SDS-PAGE. Following dialysis against 25 mM Tris-HCl, pH 8.0, 100 mM NaCl and 5% glycerol, the protein sample was loaded onto a heparin column (GE Healthcare Life Science) that had been equilibrated with the same buffer. Proteins were eluted using an NaCl step gradient, with the L10(L12)_4_ complex eluting between 450 and 550 mM NaCl, as determined by SDS-PAGE. Pure L10(L12)_4_ samples were pooled and dialyzed against 30 mM Tris-HCl, pH 7.6, 200 mM NaCl and 45% glycerol and then stored at –20°C.

### Primer extension assay

Total cellular RNA was isolated from a mid-exponential phase culture of strain PLBS338 grown in minimal-ACH medium using the RNeasy kit (Qiagen). Total RNA (5 μg/μl) was hybridized to 150 nM ^32^P end-labeled DNA oligonucleotide complementary to nt +38 to +57 relative to the start of *rplJL* transcription by heating in TE buffer to 80°C for 3 min and cooling to room temperature. Reaction mixtures (10 μl) containing 2 μl of the hybridization mixture, 375 μM of each dNTP, 10 mM DTT, 200 μg/ml BSA (Promega), 1X SuperScript III buffer and 5 Units SuperScript III Reverse Transcriptase (Life Technologies) were incubated at 42°C for 15 min. Reactions were terminated by the addition of 10 μl of stop solution (95% formamide, 20 mM EDTA, 0.025% sodium dodecyl sulfate, 0.025% xylene cyanol and 0.025% bromophenol blue). Samples were fractionated through standard 6% polyacrylamide sequencing gels. Sequencing reactions were performed using plasmid pYH134 as template and the same end-labeled DNA oligonucleotide as a primer. Radiolabeled bands were imaged on a Typhoon 8600 Variable Mode Imager (Molecular Dynamics).

### β-galactosidase assay

*B. subtilis* cultures containing *rplJ’-’lacZ* translational, *rplJ-lacZ* transcriptional or *rplLP’-’lacZ* translational fusions were grown at 37°C in LB with 1 mM IPTG. When appropriate, growth media also contained 5 μg/ml chloramphenicol, 12.5 μg/ml kanamycin or 12.5 μg/ml tetracycline. Cells were grown until mid-exponential or stationary phase and β-galactosidase activity was determined as described previously (27,28).

### Filter binding assay

Filter binding reactions followed published procedures (21,29). *rplJL* leader RNA was synthesized *in vitro* with the Agilent Technologies kit. The PCR-derived DNA template contained a T7 promoter and *rplJL-*specific sequences (+13 to +118 relative to *rplJL* transcription). Gel-purified RNAs were dephosphorylated with calf intestinal alkaline phosphatase and then 5′ end-labeled with [γ-^32^P]ATP and T4 polynucleotide kinase (New England Biolabs). Reaction mixtures (50 μl) contained 10% glycerol, 0.2 mg/ml yeast RNA, 50 μg/ml BSA, 0.01 nM labeled RNA and various concentrations of purified L10(L12)_4_ in binding buffer (30 mM Tris-HCl, pH 7.6, 175 mM KCl and 3 mM MgCl_2_). Reactions were incubated at 37°C for 30 min prior to filtration through 0.2 μm nitrocellulose (Whatman) and Hybond-N+ (Amersham) membranes using a PR 600 Slot-Blot Filtration Manifold (Amersham Biosciences). After filtering, wells were washed twice with 200 μl of binding buffer. Membranes were dried and imaged on a Typhoon 8600 Variable Mode Imager and quantified using Imagequant 5.2 (Molecular Dynamics). For a given protein concentration, values corresponding to the fraction of bound RNA were averaged and standard deviations were calculated from four independent experiments performed in duplicate. The fraction of RNA bound was plotted as a function of L10(L12)_4_ concentration using Kaleidagraph 3.6 (Synergy Software) and fit to the binding equation f_bound_ = f_max_ [L10(L12)_4_] / ([L10(L12)_4_] + *K*d); where *K*d is the dissociation binding constant and f_max_ is the maximum fraction of bound RNA. Fits were weighted to the standard deviation of each data point.

### Footprint assay

5′ end-labeled RNA (+13 to +118 relative to *rplJL* transcription) was generated as described above. In-line probing reactions were performed to optimize the incubation time to prevent multiple cleavages in any one transcript. Binding reactions (10 μl) containing 50 mM Tris-HCl, pH 8.3, 20 mM MgCl_2_, 100 mM KCl, 0.2 mg/ml yeast RNA, 15% glycerol, 200 μg/ml BSA, 1 nM *rplJL* leader RNA and various concentrations of L10(L12)_4_. In-line probing reactions were incubated at 25°C for 40 h (30). Reactions were terminated by adding an equal volume of the same stop solution used in the primer extension analysis. Partial alkaline hydrolysis and RNase T1 digestion ladders were prepared as described previously (31). Samples were fractionated through 6% denaturing polyacrylamide gels and radiolabeled bands were imaged on a Typhoon 8600 Variable Mode Imager.

### Boundary analysis

3′ boundary analysis was carried out by modifying previously published procedures (31,32). To determine the 3′ boundary required for L10(L12)_4_ binding, 5′ end-labeled RNA (+13 to +118 relative to *rplJL* transcription) was generated as described above. To generate 5′ end-labeled alkaline hydrolysis ladders, 50-μl RNA samples (10 pmol) were incubated at 95°C for 4 min in alkaline hydrolysis buffer (100 mM NaHCO_3_/Na_2_CO_3_, pH 9.0 and 2 mM EDTA) and then recovered by ethanol precipitation. Hydrolyzed RNAs were mixed with 1 or 3 μM L10(L12)_4_ (20 μl reaction volume) and incubated at 37°C for 30 min to allow L10(L12)_4_-RNA complex formation. Samples were fractionated through 10% native polyacrylamide gels. Bound and unbound transcripts were visualized by autoradiography, excised from the gel and subsequently eluted from the gel. RNAs were ethanol precipitated and fractionated through 6% denaturing polyacrylamide gels. RNase T1 digestion and alkaline hydrolysis ladders of the same transcript were used as molecular size standards.

### *In vitro* transcription assay

Multi-round *in vitro* transcription reactions were performed by modifying a published procedure (33). His-tagged NusA (34), His-tagged NusG (35) and His-tagged *B. subtilis* Eσ^A^ RNAP (36,37) were purified as described previously. Transcription reaction mixtures (6 μl) contained 40 mM Tris-HCl, pH 8.0, 80 mM KCl, 4 mM MgCl_2_, 4% trehalose, 5 mM DTT, 0.27 mM ATP, 0.07 mM CTP, 0.11 mM GTP, 0.11 mM UTP, 0.1 μg/μl RNAP, 20 nM template DNA, 200 μg/ml BSA and 0.36 μl [α^32^P]UTP (3000Ci/mmol). When used, oligonucleotides complementary to the 5′ portion of the AAT, AT or T structures were added at 20 μM (1000-fold excess over template DNA). Reaction mixtures were incubated at 30°C for 20 min and were stopped by adding an equal volume of the stop solution used in the primer extension assay. Samples were fractionated through 6% polyacrylamide sequencing gels. The PCR-derived DNA template used in this analysis contained –382 to +200 relative to the start of *rplJL* transcription.

## RESULTS

### RNA structure modeling of the *rplJL* operon leader

**Figure 1.**
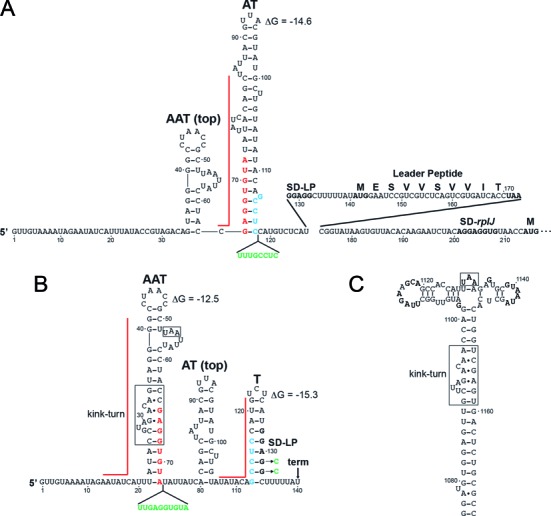
Autogenous *rplJL* transcription attenuation model. (**A**) In the absence of bound L10(L12)_4_ the antiterminator structure (AT) will form, resulting in transcription readthrough. Translation of the leader peptide of readthrough transcripts presumably prevents Rho-dependent termination further downstream. (**B**) Bound L10(L12)_4_ stabilizes the anti-antiterminator structure (AAT), thereby promoting formation of the terminator hairpin (T) and termination at the position indicated by the arrow (term). (**A**, **B**) The thermodynamic stability of the AAT, AT and T structures predicted by MFOLD are shown. Overlapping residues between the AAT and AT structures are in red, while overlapping residues between the AT and T structures are in cyan. The top portions of the AAT and AT structures are shown as a point of reference. Positions of the Shine–Dalgarno sequence for the leader peptide (SD-LP) and *rplJ* (SD-*rplJ*), as well as the corresponding translation initiation and stop codons are in bold type. Red lines represent regions of the AAT, AT and T structures that hybridize to complementary oligonucleotides used for *in vitro* transcription studies. Mutations that render the AAT, AT or T structures nonfunctional are in green. Numbering is relative to the start of transcription. (**C**) L10(L12)_4_ binding target in 23S rRNA. (**B**, **C**) The conserved kink-turn motif and UAA sequence (boxed) involved in L10(L12)_4_ binding to the AAT and 23S rRNA are shown.

The ribosomal *rplJL* operon from *E. coli* is regulated by a translation repression mechanism in which the encoded L10(L12)_4_ complex binds to a secondary structure that forms in its mRNA upstream of the *rplJ* coding sequence (22,23). Studies with the L10(L12)_4_ complex from *G. stearothermophilus* showed that the recognition hairpin within the *rplJL* leader transcript functions as a molecular mimic of its 23S rRNA binding site (21). Based on the structural similarity of the predicted structures in several bacterial species including *E. coli, G. stearothermophilus* and *B. subtilis*, it was proposed that the *rplJL* operons from each organism would be regulated by a similar translation repression mechanism (21). However, a bioinformatics study identified potential overlapping RNA structures in the *B. subtilis rplJL* leader, suggesting that regulation could occur by a transcription attenuation mechanism (38). RNA structure predictions using MFOLD (39) allowed us to identify three overlapping RNA structures in the *B. subtilis rplJL* leader (Figure 1). The promoter proximal structure, which would have the first opportunity to form, shares 8 nts in common with a second structure immediately downstream, indicating that their formation would be mutually exclusive. This second structure, in turn, shares 5 nts in common with the third promoter distal structure, again making their formation mutually exclusive. This third structure was followed by a U-rich sequence, suggesting that it would function as an intrinsic transcription terminator (T). Therefore, the second structure could function as an antiterminator (AT), while the first structure could serve as an anti-antiterminator (AAT). Notably, the putative AAT structure contains the L10(L12)_4_ binding target, including a kink-turn motif and a UAA sequence shown to be critical for *G. stearothermophilus* L10(L12)_4_-RNA interaction (21). The three overlapping structures that we identified were consistent with a transcription attenuation model in which L10(L12)_4_ binding would favor formation of the AAT structure, thereby preventing formation of the AT, and hence promoting transcription termination. In the absence of L10(L12)_4_ binding, formation of the AT structure would allow transcription readthrough into the *rplJL* structural genes. We performed experiments to determine whether the L10(L12)_4_ complex from *B. subtilis* regulates its expression and to identify the underlying regulatory mechanism.

### L10(L12)_4_ represses expression of the *rplJL* operon

**Figure 2.**
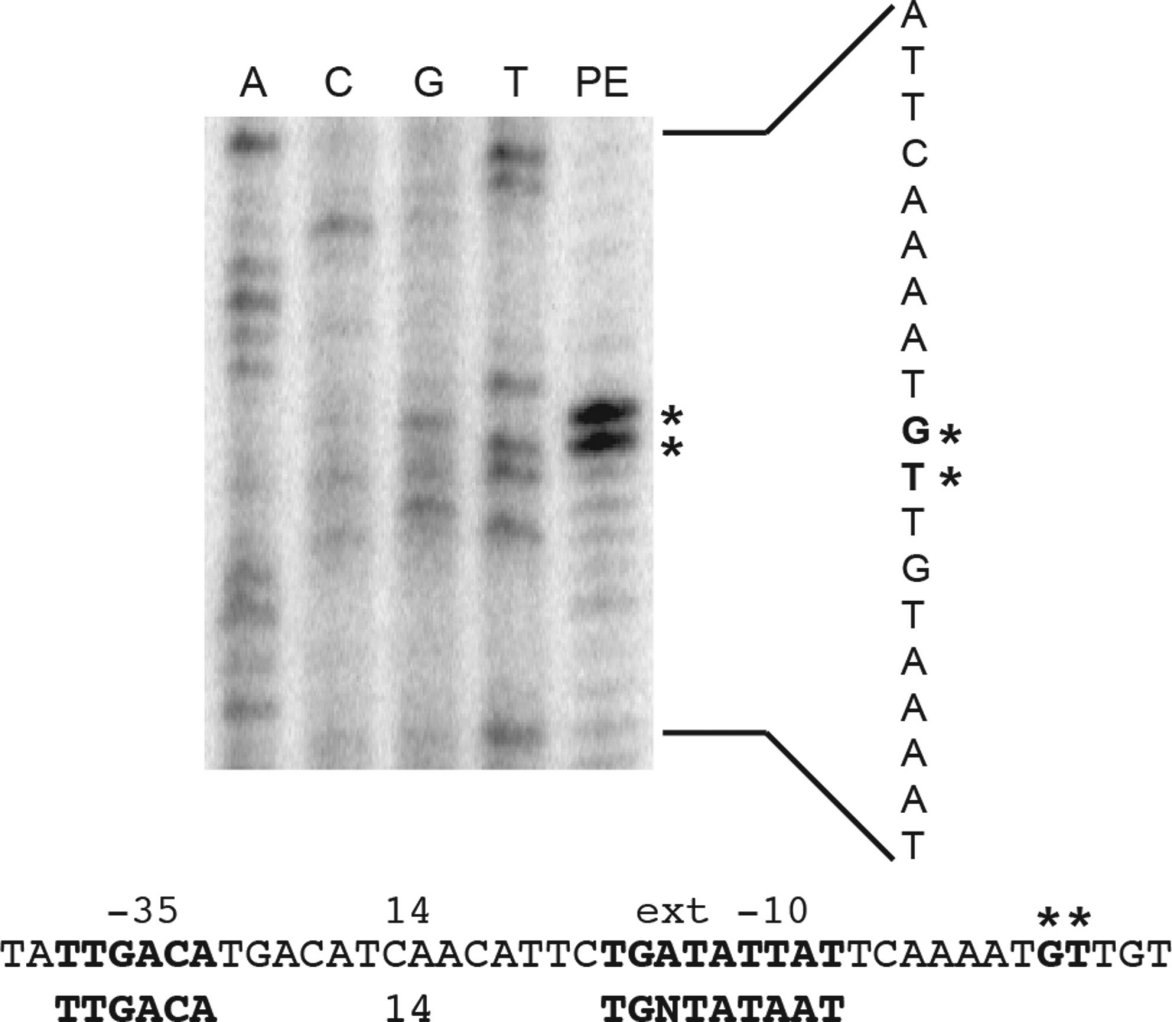
Identification of the *rplJL* transcription start site and a σ^A^-dependent promoter. Primer extension (PE) was used to map of the 5′ end of the *rplJL* transcript. Asterisks mark the two PE products that correspond to the 5′ end of transcripts originating from the *rplJL* promoter. The sequence of the σ^A^-dependent *rplJL* promoter (−35 and extended −10), as well as the σ^A^ consensus promoter is shown. The transcription start sites are shown in bold type and marked by asterisks.

Primer extension experiments were carried out using total cellular RNA from an exponential phase culture to map the 5′ end of *rplJL* operon transcripts. Two adjacent primer extension products corresponding to G and U residues in the mRNA were observed just downstream from a near consensus σ^A^-dependent promoter containing an extended −10 sequence (Figure 2). These results indicate that a 213-nt leader precedes the *rplJ* coding sequence, including sequences involved in formation of the predicted AAT, AT and T structures (Figure 1).

**Figure 3.**
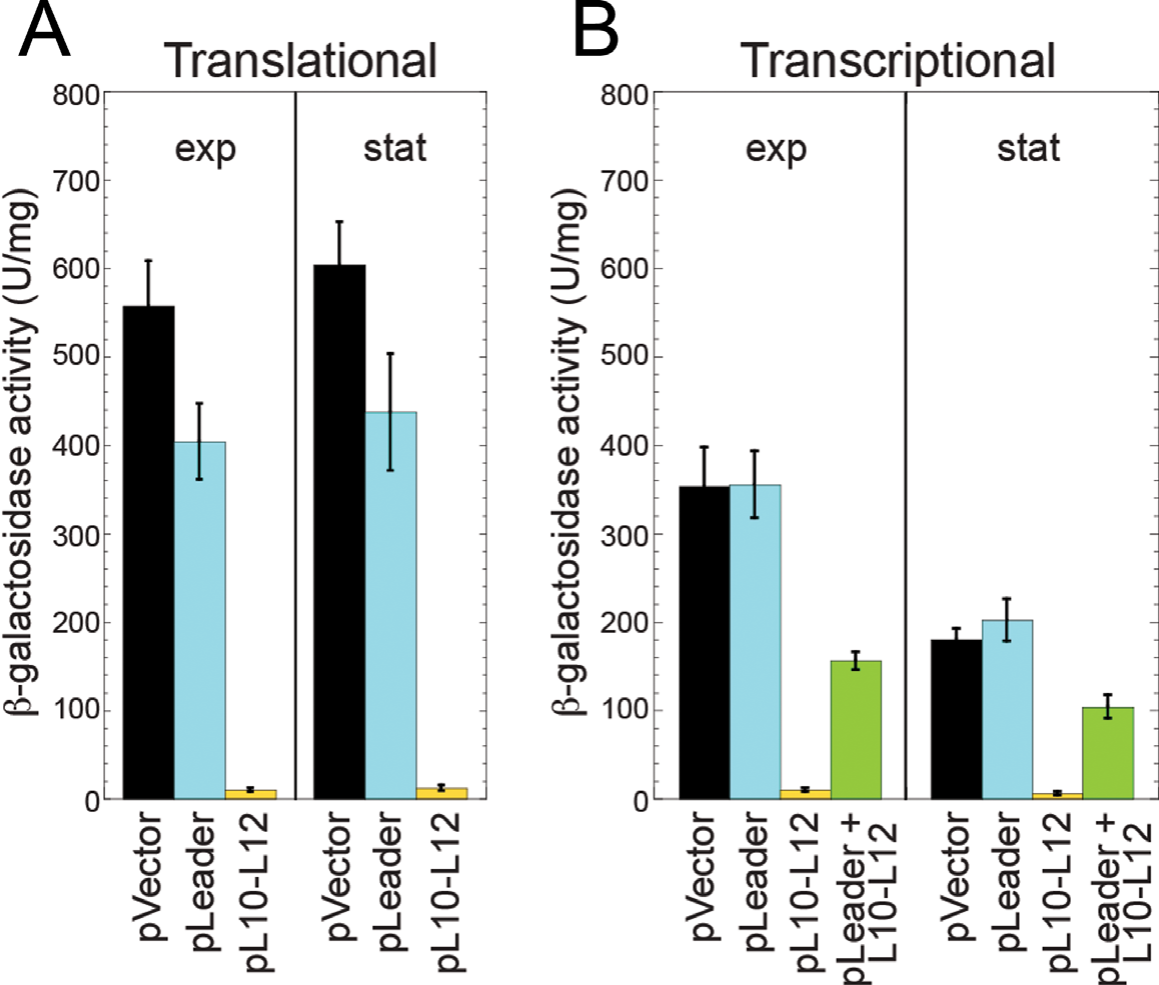
*rplJL* expression is regulated by L10(L12)_4_. Strains contained chromosomally integrated *rplJ’-’lacZ* translational (**A**) or *rplJ-lacZ* transcriptional (**B**) fusions. Cultures were harvested in exponential (exp) or stationary (stat) phase and β-galactosidase activity was determined. Error bars reflect the standard error of at least three independent experiments. Each strain contained either an empty vector control plasmid (pVector), a plasmid expressing the *rplJL* leader (pLeader), a plasmid expressing leaderless *rplJL* (pL10–L12), or a plasmid separately expressing the *rplJL* leader and leaderless *rplJL* (pLeader + L10–L12).

An *rplJ’-’lacZ* translational fusion containing nucleotides −382 to +231 relative to the start of *rplJL* transcription driven by the *rplJL* promoter was constructed and subsequently integrated into the *amyE* locus of the *B. subtilis* chromosome (Figure 1). Expression from the translational fusion was similar in exponential and stationary phase cultures (Figure 3a). Plasmid pL10–L12 containing an IPTG-inducible promoter driving expression of *rplJL* without the leader sequence was introduced into this strain to determine whether L10(L12)_4_ regulated its expression. Overexpression of *rplJL* greatly reduced expression of the translational fusion (Figure 3a). We also introduced a plasmid containing the same IPTG-inducible promoter expressing the *rplJL* leader without the protein coding sequence (pLeader). Although we expected that overexpression of the leader from this plasmid would titrate out L10(L12)_4_ and relieve autogenous repression, this outcome was not observed (Figure 3a). Expression studies were also carried out with an integrated *rplJ-lacZ* transcriptional fusion containing nucleotides −382 to +200 relative to the start of *rplJL* transcription (Figure 1). The results obtained with this fusion were similar to the translational fusion except that expression was about 2-fold higher in exponential phase compared to stationary phase (Figure 3b). As the results with the translational and transcriptional fusions were comparable, we conclude that L10(L12)_4_ represses its expression at the transcriptional level.

The plasmid expressing the *rplJL* leader did not relieve L10(L12)_4_-mediated repression of either fusion. The most likely explanation for this result is that expression of the fusion was already fully derepressed under our growth conditions, i.e. sufficient levels of 23S rRNA were available to bind most of the L10(L12)_4_ complexes for ribosome biogenesis. To test this possibility we moved the IPTG-inducible *rplJL* leader construct into the same plasmid that overexpressed L10(L12)_4_, and subsequently introduced the resulting plasmid into the transcriptional fusion strain. In this case, overexpression of the *rplJL* leader relieved autogenous L10(L12)_4_-mediated repression, indicating that the *rplJL* leader transcript is capable of titrating out excess L10(L12)_4_ (Figure 3b).

### L10(L12)_4_ binds to the predicted AAT structure

The L10(L12)_4_ complex was purified using a His6 tag on the N-terminus of L10. We performed filter binding assays using an *rplJL* leader transcript extending from +13 to +118 and yeast RNA as a nonspecific competitor. Non-linear least-squares analysis of the data from four independent experiments yielded an apparent *K*d of 33 ± 10 nM.

**Figure 4.**
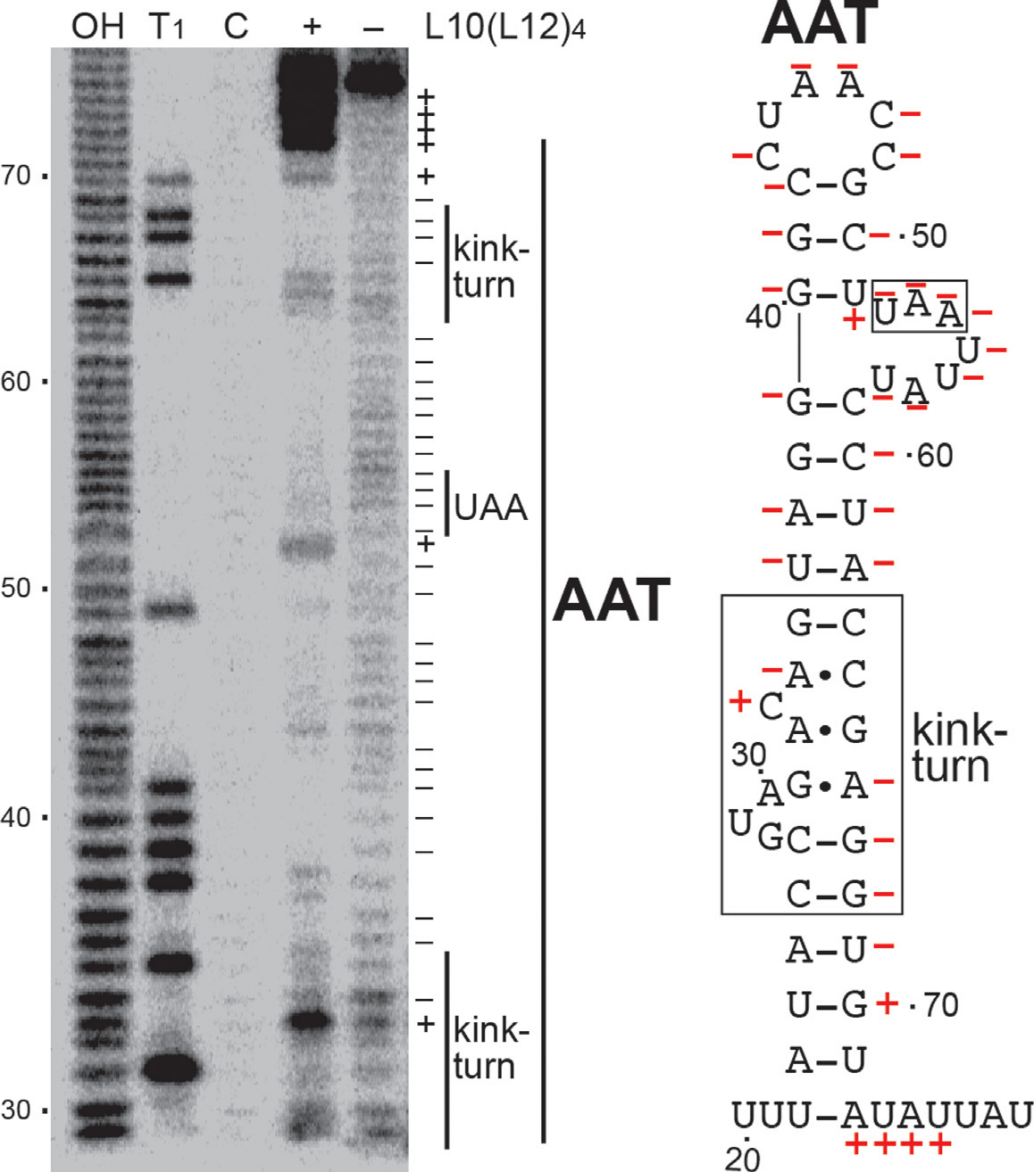
L10(L12)_4_-*rplJL* leader RNA footprint. In-line probing was carried out in the absence or presence of 3 μM L10(L12)_4_. Partial alkaline hydrolysis (OH) and RNase T1 digestion (T1) ladders, as well as control (C) lanes in the absence of in-line treatment are shown. The RNase T1 ladder was generated under denaturing conditions so that every G residue in the transcript could be visualized. Residues in which in-line cleavage was reduced (–) or increased (+) in the presence of L10(L12)_4_ are marked on the right of the gel and on the AAT structure. The conserved kink-turn motif (boxed) and UAA sequence (boxed) involved in L10(L12)_4_ binding to the AAT are shown. Numbering is relative to the start of transcription.

Having established specific interaction between L10(L12)_4_ and *rplJL* leader RNA, L10(L12)_4_–*rplJL* leader RNA footprint experiments were carried out using in-line probing (Figure 4). This method makes use of the inherent instability of flexible single-stranded RNA segments (30). The RNA used for this analysis extended from +13 to +118, including all of the residues involved in formation of the predicted AAT and AT structures. In the absence of L10(L12)_4_, essentially every nucleotide predicted to be part of the AAT structure was cleaved with comparable efficiency, suggesting that this RNA segment was relatively flexible and devoid of stable structure. However, addition of the L10(L12)_4_ complex caused reduced cleavage of several regions of the RNA, including predicted base-paired regions and several single-stranded residues. These single-stranded residues correspond to the conserved UAA motif previously shown to be important for *G. stearothermophilus* L10(L12)_4_ interaction with the *rplJL* leader transcript (21), as well as several residues in the adjacent loop at the apex of the AAT structure. From these data we conclude that bound L10(L12)_4_ stabilizes the AAT structure and protects several single-stranded residues from in-line attack.

**Figure 5.**
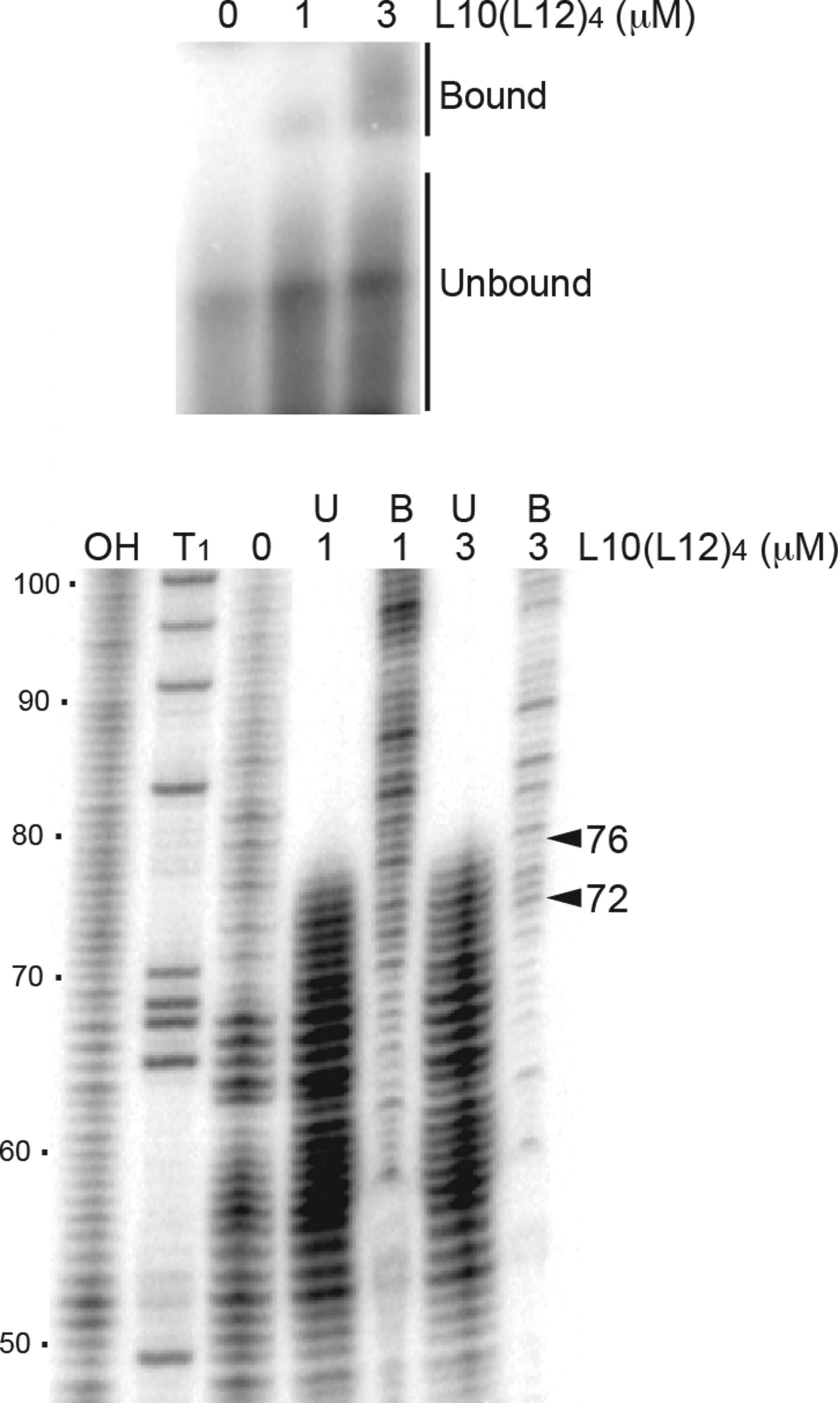
3′ boundary analysis of L10(L12)_4_-*rplJL* leader RNA interaction. A limited alkaline hydrolysis ladder of 5′ end-labeled *rplJL* leader RNA was incubated with 1 or 3 μM L10(L12)_4_. Bound L10(L12)_4_-RNA complexes were separated from unbound RNA on a native gel (top panel) and subsequently fractionated through a 6% denaturing gel (bottom panel). Lanes corresponding to limited alkaline hydrolysis (OH) and RNase T1 digestion (T1) ladders are marked. The arrowheads between positions 72 and 76 demark the boundary between bound (B) and unbound (U) RNA. Numbering is relative to the start of transcription.

A 3′ boundary analysis was also performed to more precisely define the extent of the AAT structure required for L10(L12)_4_ binding (Figure 5). RNAs were 5′ end-labeled, hydrolyzed to obtain a ladder of 5′ end-labeled transcripts and subsequently mixed with L10(L12)_4_. Bound and unbound RNAs were separated by native gel electrophoresis, gel purified and then separated on a standard denaturing sequencing gel. The transition between bound and unbound RNA was relatively sharp. All transcripts extending to position 76 and beyond were bound by L10(L12)_4_, while binding was greatly reduced or absent for transcripts that did not extend to position 72. As the 3′ end of the AAT structure extends to position 72 (Figure 1B), we conclude that the entire structure is important for tight L10(L12)_4_ binding.

### L10(L12)_4_ promotes transcription termination in the *rplJL* leader region

**Figure 6.**
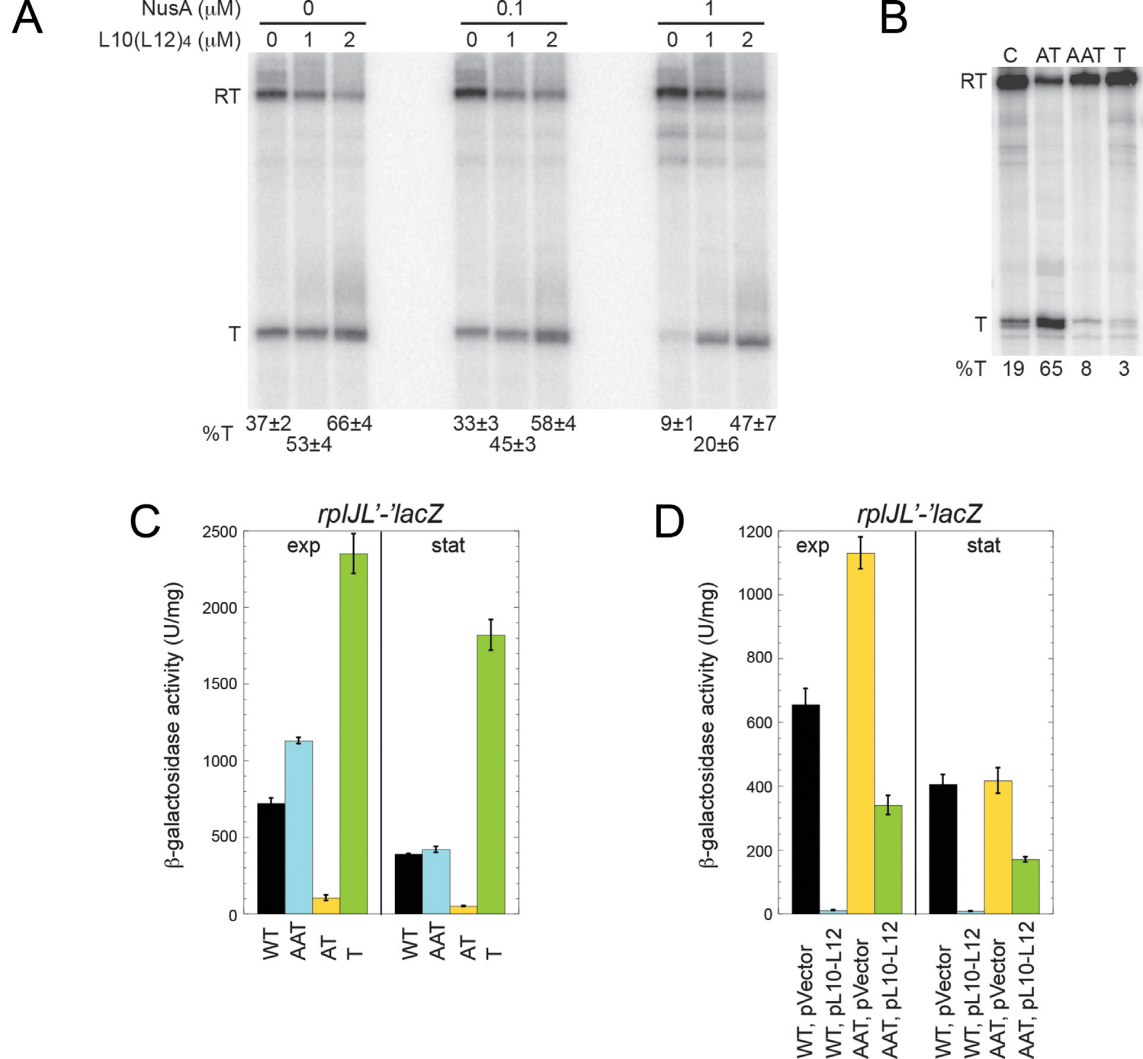
L10(L12)_4_ regulates *rplJL* by an autogenous transcription attenuation mechanism. (**A**) *In vitro* transcription reactions were performed in the presence of the indicated concentration of L10(L12)_4_ and/or NusA and the resulting transcripts were fractionated in denaturing 6% polyacrylamide gels. Positions of terminated (T) and readthrough (RT) transcripts are indicated. A representative gel is shown with quantitation of termination efficiency (%T) from at least 5 gels shown below each lane. (**B**) *In vitro* transcription reactions containing 0.1 μM NusA were performed in the presence of an oligonucleotide complementary to the 5′ side of the AAT, AT or T structures. An oligonucleotide that was not complementary to any portion of the *rplJL* leader was used as a control (C). Positions of terminated (T) and readthrough (RT) transcripts are indicated. %T is shown at the bottom of each lane. A representative gel is shown with quantitation of at least 10 gels shown in Table 1. (**C**) Effect of mutations that render the AAT, AT or T structures nonfunctional (Figure 1). Strains contained integrated wild type (WT) or mutant *rplJ’-’lacZ* translational fusions. (**D**) Effect of a mutation that renders the AAT structure nonfunctional (Figure 1). Strains contained integrated WT or mutant *rplJ’-’lacZ* translational fusions. Each strain also contained either an empty vector control plasmid (pVector) or a plasmid expressing leaderless *rplJL* (pL10–L12). (C,D) Cultures were harvested in exponential (exp) or stationary (stat) phase and β-galactosidase activity was determined. Error bars reflect the standard error of at least three independent experiments.

Our model predicted that L10(L12)_4_ binding to the AAT structure would promote transcription termination by preventing formation of the AT structure (Figure 1). *In vitro* transcription reactions were carried out to determine whether L10(L12)_4_ affected the level of termination in the *rplJL* leader region. Because NusA and NusG are general transcription elongation factors, we also examined the effects of these proteins in our *in vitro* transcription assay. As NusG did not influence the level of termination under any condition that we tested (data not shown), this protein was not included in any of the transcription reactions described herein. In the absence or presence of 0.1 μM NusA, L10(L12)_4_ led to a modest increase in termination. However, in the presence of 1 μM NusA, L10(L12)_4_ caused a 5-fold increase in termination (Figure 6a).

A central feature of the proposed model of the *rplJL* transcription attenuation mechanism is that formation of the AAT structure would prevent formation of the AT structure, resulting in increased termination. In contrast, formation of the AT structure would promote transcription readthrough into the *rplJL* structural genes (Figure 1). To test these aspects of the model, DNA oligonucleotides that are complementary to the 5′ side of the AAT, AT or T structures were added separately to the *in vitro* attenuation assay to determine whether they would affect the extent of termination. We predicted that disruption of either the AAT or T structures by a complementary DNA oligonucleotide would decrease termination. Conversely, disruption of the AT structure by a complementary oligonucleotide would increase termination (Figure 1). Each oligonucleotide was added to the transcription attenuation assay in 1000-fold molar excess over template DNA. As predicted, the oligonucleotide complementary to the AT structure resulted in increased termination, particularly in the presence of 1 μM NusA (Figure 6b and Table 1). Similarly, oligonucleotides complementary to the AAT or T structures resulted in decreased termination, particularly in the presence of 0 or 0.1 μM NusA (Figure 6b and Table 1).

**Table 1.** Effect of oligonucleotide competitors on termination in the *rplJL* leader

	Termination (%)		
Oligonucleotide	0 μM NusA	0.1 μM NusA	1 μM NusA
None	41 ± 2	30 ± 2	12 ± 3
Control	36 ± 2	26 ± 2	12 ± 1
AT	50 ± 1	57 ± 3	63 ± 2
AAT	6 ± 1	7 ± 1	16 ± 2
T	3 ± 0.6	3 ± 0.5	3 ± 0.6

*In vitro* transcription reactions were performed in the presence of the indicated concentration of NusA and an oligonucleotide complementary to the 5′ side of the antiterminator (AT), anti-antiterminator (AAT) or terminator (T) structures (Figure 1). The control oligonucleotide was not complementary to any portion of the *rplJL* 5′ UTR. Termination values are averages of at least 10 independent experiments ± standard deviation.

As a further test of the attenuation model, we introduced mutations into the *rplJL* leader that were predicted to render the AAT, AT or T nonfunctional. Rather than introduction of point mutations into the AAT and AT structures that could lead to unintended RNA structural rearrangements, we introduced insertions that repeated the overlapping sequences between these structures. Thus, one mutation was predicted to inactivate AAT function by allowing simultaneous formation of the AAT and AT structures separated by two U residues (Figure 1b), whereas another mutation was predicted to inactivate AT function by allowing formation of both the AT and T structures with a three nucleotide separation (Figure 1a). We found that the AT mutation resulted in a substantial reduction in expression of the *rplJ’-’lacZ* translational fusion as expected, whereas the AAT mutation only resulted in a small increase in expression (Figure 6c). In the case of the T mutation (Figure 1b), we disrupted the base of the terminator hairpin by introducing two mismatches in the hairpin. MFOLD prediction suggested that these changes would prevent formation of three base pairs at the bottom of the hairpin, thereby shortening the terminator hairpin and simultaneously introducing three non-U residues into the downstream U-tract. As expected, this mutation resulted in increased *rplJ’-’lacZ* expression (Figure 6c). The finding that inactivation of AAT function had little effect on expression is consistent with our previous findings that *rplJL* expression was already derepressed under our growth conditions (Figure 3). Thus we tested the effect of this mutation in the presence of the plasmid that overexpresses *rplJL* (pL10–L12). In this case, inactivating AAT function by allowing simultaneous formation of the AAT and AT structures led to a dramatic increase in expression (Figure 6d, compare WT with AAT). From these data we conclude that the AAT, AT and T structures function as predicted and that L10(L12)_4_ autoregulates expression of the *B. subtilis rplJL* operon by transcription attenuation.

### Translation of an *rplJL* leader peptide increases expression of the *rplJL* operon

**Figure 7.**
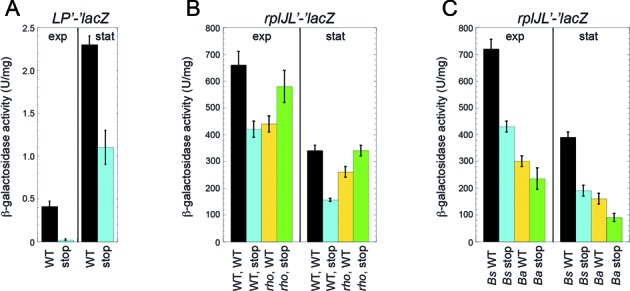
Expression of a leader peptide controls *rplJL* expression by an antitermination mechanism. (**A**) Strains contained an integrated wild type (WT) *LP’-’lacZ* translational fusion or one in which the leader peptide start codon was converted to a stop codon (stop). (**B**) WT or *rho* mutant strains contained an integrated WT *rplJ’-’lacZ* translational fusion or one in which the leader peptide start codon was converted to a stop codon (stop). (**C**) Strains contained an integrated *rplJ’-’lacZ* translational fusion with the WT *B. subtilis* (*Bs*) or *B. amyloliquefaciens* (*Ba*) leader peptide coding sequence or one in which the leader peptide start codon was converted to a stop codon (stop). (A,B,C) Cultures were harvested in exponential (exp) or stationary (stat) phase and β-galactosidase activity was determined. Error bars reflect the standard error of at least three independent experiments.

Inspection of the *rplJL* leader region revealed a possible leader peptide (LP) coding sequence whose SD sequence is within the 3′ portion of the terminator hairpin (Figure 1). However, if the antiterminator formed and RNAP transcribed past the intrinsic termination signal, the SD sequence would be single-stranded and available for ribosome binding (Figure 1a). We tested whether the leader peptide was expressed by generating an *rplLP’-lacZ* translational fusion containing nucleotides −382 to +170 relative to the start of *rplJL* transcription (Figure 1) in which the LP stop codon was replaced with the *lacZ* coding sequence. Although expression from this fusion was very low in exponential phase, it increased in early stationary phase (Figure 7a). We also found that changing the LP start codon to a stop codon eliminated expression of the leader peptide, although we observed higher background levels in early stationary phase.

We next tested whether translation of the LP affected expression of the *rplJ’-’lacZ* translational fusion by replacing the LP start codon with a stop codon. Introduction of the LP stop codon reduced expression of the fusion, indicating that translation of the LP increases *rplJL* expression (Figure 7b). Since the LP coding sequence is downstream from the intrinsic terminator it seemed unlikely that the LP affected the L10(L12)_4_–mediated attenuation mechanism. Instead, we considered the possibility that expression of the LP would reduce Rho-dependent termination. To test this possibility, we determined the effect of deleting *rho* on expression of the WT and LP stop codon *rplJ’-’lacZ* translational fusions. Deletion of *rho* completely suppressed the expression defect caused by the LP stop codon mutation (Figure 7b, compare WT stop with *rho* stop). These data indicate that translation of the LP increases expression of the *rplJL* operon about 2-fold, presumably by blocking Rho access to the nascent *rplJL* transcript.

We also replaced the *B. subtilis* LP sequence with a shorter version from *B. amyloliquefaciens* that also differed considerably in its amino acid sequence (Supplementary Figure S1). Expression of this LP also increased expression of the *rplJL’-’lacZ* fusion but not as effectively as the natural *B. subtilis* sequence (Figure 7c, compare WT with stop codon mutations), suggesting that the length and/or sequence of the LP may contribute to this antitermination mechanism.

## DISCUSSION

Our motivation for exploring the regulatory mechanism controlling *B. subtilis rplJL* expression was 2-fold. Although much is known about the mechanisms used by *E. coli* to control ribosomal protein gene expression, comparatively little is known about the mechanisms controlling these genes in *B. subtilis*. Moreover, previous studies led to the suggestion that *B. subtilis rplJL* expression would be controlled by a translation repression mechanism (21), whereas a bioinformatics study suggested that expression of this *B. subtilis* operon might be controlled by transcription attenuation (38). Our studies demonstrate that the *B. subtilis rplJL* operon is autoregulated by a transcription attenuation mechanism in which bound L10(L12)_4_ stabilizes an AAT structure, thereby preventing formation of the AT, and hence promoting transcription termination. In the absence of bound L10(L12)_4_, formation of the AT structure allows transcription into the *rplJL* structural genes (Figure 1).

Previous studies demonstrated that a kink-turn motif and a UAA sequence within a secondary structure were critical components of the L10(L12)_4_ recognition target (21). Results from our footprinting (Figure 4) and boundary (Figure 5) studies extend this information by showing that L10(L12)_4_ stabilizes the recognition hairpin and that the entire structure is required for tight binding.

NusA is an essential general transcription elongation factor that has a variety of activities in different contexts (40,41). NusA from *B. subtilis* and/or *E. coli* is known to stimulate hairpin-dependent pausing (34,42), increase intrinsic termination (34,41), stimulate phage HK022 Nun-mediated transcription arrest (43), is a component of rRNA and λN antitermination complexes (44), participates in transcription-coupled DNA repair (45) and promotes cotranscriptional folding of the RNA component of RNase P (46). Thus, this elongation factor has a variety of known functions, and almost certainly some that have yet to be discovered. Although our *in vitro* transcription results show that NusA inhibits termination in the *rplJL* 5′ UTR (Figure 6), we feel it is unlikely that it does so by interfering with termination *per se*. Instead, we favor a model in which NusA promotes formation of the AT structure, perhaps by stimulating RNAP pausing before RNAP transcribes the terminator sequence.

In addition to the L10(L12)_4_-dependent attenuation mechanism, we found that translation of a leader peptide increases expression of the *rplJL* operon, presumably by inhibiting Rho access to the nascent transcript and thereby reducing Rho-dependent termination (Figures 1 and 7). Although expression of the leader peptide is low, it leads to a substantial increase in expression of the *rplJL* operon. How might low-level leader peptide translation have such a profound effect on *rplJL* expression? A single round of leader peptide translation would be sufficient to block Rho access to the nascent *rplJL* transcript. Since every transcript that escapes Rho-dependent termination can be translated multiple times, the effect of leader peptide synthesis on *rplJL* expression would be amplified. Thus, in addition to the L10(L12)_4_-dependent attenuation mechanism, our findings suggest that the *rplJL* operon is regulated by a leader peptide-dependent antitermination mechanism. Some leader peptides are known to interact with the peptide exit channel within the ribosome, thereby causing translation to arrest (47,48). However, the hydrophobic valine-rich *rplJL* leader peptide does not contain a critical proline residue present in most of the leader peptides that have been shown to cause translation arrest in bacteria (47). Nevertheless, the finding that the *B. amyloliquefaciens* LP sequence is less effective that the natural *B. subtilis* sequence suggests that the length and/or sequence of the LP may contribute to this antitermination mechanism (Figure 7c and Supplementary Figure S1).

It is apparent that both of these regulatory mechanisms are conserved in several *Bacillus* species. *B. subtilis, B. atrophaeus, B. amyloliquefaciens, B. licheniformis, B. pumilus, B. anthracis* and *B. kaustophilus* all have similar overlapping AAT, AT and T structures that precede the *rplJ* coding sequence (Figure 1 and not shown). Furthermore, a leader peptide coding sequence is present in each organism (Supplementary Figure S1), with the SD sequence in the 3′ portion of the terminator hairpin as shown for *B. subtilis* (Figure 1). With the exception of *B. anthracis* and *B. kaustophilus*, a similar antitermination mechanism might occur as we determined for *B. subtilis*. In the case of *B. kaustophilus*, the leader peptide overlaps the *rplJ* coding sequence by 17 nucleotides, suggesting that the leader peptide and *rplJ* are translationally coupled. Note that this extensive overlap is similar to the 29 nucleotide overlap between the translationally coupled *B. subtilis trpE* and *trpD* genes (49). In contrast, the *B. anthracis* leader peptide coding sequence is in frame with *rplJ*, and its translation would result in a 16 amino acid N-terminal extension on L10.

It was previously reported that ribosomal protein L20, which is encoded by *rplT*, autogenously controls expression of the *B. subtilis infC-rpmI-rplT* operon by transcription attenuation (16). Thus the *rplJL* operon constitutes the second example of an attenuation mechanism controlling expression of ribosomal protein genes in *B. subtilis*. The finding that *rplJL* is also controlled by an apparent antitermination mechanism indicates that expression of this operon is controlled by both intrinsic and Rho-dependent termination. These dual post-transcriptional control mechanisms may allow fine-tuning of *rplJL* expression depending on the growth status of the cell. It is interesting to note that both of these operons are controlled by translation repression mechanisms in *E. coli* (22,23,50,51), although the implication of this distinction is not clear as both of these organisms autogenously control the ribosomal protein genes *rpsO* and *rpsD* at the translational level (17,19,20,52).

## SUPPLEMENTARY DATA

Supplementary Data are available at NAR Online.

SUPPLEMENTARY DATA
