# Supplementary material for: Ribosomal protein L10(L12)4 autoregulates expression of the Bacillus subtilis rplJL operon by a transcription attenuation mechanism
Source: Nucleic Acids Res. 2015 Jun 22;43(14):7032–43. doi: 10.1093/nar/gkv628 (PMC4538822; doi:10.1093/nar/gkv628)
Supplement: SUPPLEMENTARY DATA [file supp_43_14_7032__index.html]

Ribosomal protein L10(L12)4 autoregulates expression of the Bacillus subtilis rplJL operon by a transcription attenuation mechanism — Ribosomal protein L10(L12)4 autoregulates expression of the Bacillus subtilis rplJL operon by a transcription attenuation mechanism — SUPPLEMENTARY DATA 

# Ribosomal protein L10(L12)4 autoregulates expression of the *Bacillus subtilis rplJL* operon by a transcription attenuation mechanism

## SUPPLEMENTARY DATA

- SUPPLEMENTARY DATA
