# Supplementary material for: Ribosomal protein L10(L12)4 autoregulates expression of the Bacillus subtilis rplJL operon by a transcription attenuation mechanism
Source: Nucleic Acids Res. 2015 Jun 22;43(14):7032–43. doi: 10.1093/nar/gkv628 (PMC4538822; doi:10.1093/nar/gkv628)
Supplement: SUPPLEMENTARY DATA [file supp_gkv628_nar-01301-r-2015-File010.pdf]

Supplementary Material

Ribosomal protein L10(L12)$_4$ autoregulates expression of the *Bacillus subtilis rplJL* operon by a transcription attenuation mechanism

Helen Yakhnin, Alexander V. Yakhnin and Paul Babitzke

Table S1. Bacterial strains

Strain	Description [a, b]	Source
PLBS338	Prototroph	1
PLBS655	PLBS338/*rplJ-lacZ*	This study
PLBS656	PLBS338/*rplJ'-'lacZ*	This study
PLBS751	PLBS655/pL10-L12	This study
PLBS752	PLBS655/pLeader	This study
PLBS753	PLBS655/pVector	This study
PLBS754	PLBS656/pL10-L12	This study
PLBS755	PLBS656/pLeader	This study
PLBS756	PLBS656/pVector	This study
PLBS767	PLBS655/pLeader + L10-L12	This study
PLBS768	PLBS338/*rplLP'-'lacZ*	This study
PLBS770	PLBS338/*rplJ'-'lacZ* (LP ATG start to TAG stop codon)	This study
PLBS772	PLBS656/*rho::neo*	This study
PLBS774	PLBS770/*rho::neo*	This study
PLBS775	PLBS338/LP$_{Ba}$-*rplJ'-'lacZ* (LP *B. amyloliquefaciens*)	This study
PLBS777	PLBS338/*rplJ'-'lacZ* (117-∇UUUGCCUC-118)	This study
PLBS778	PLBS338/*rplJ'-'lacZ* (G131C:G132C)	This study
PLBS780	PLBS338/*rplJ'-'lacZ* (72-∇UUGAGGUGUA-73)	This study
PLBS782	PLBS780/pVector	This study
PLBS783	PLBS780/pL10-L12	This study
PLBS786	PLBS338/*rplLP'-'lacZ* (LP ATG start to TAG stop codon)	This study
PLBS788	PLBS775/ LP$_{Ba}$-*rplJ'-'lacZ* (LP ATG start to TAG stop codon)	This study

[a] *rplJ-lacZ* transcriptional fusions contain nucleotides -382 to +200 relative to the start of *rplJ* transcription. *rplJ'-'lacZ* translational fusions contain nucleotides -382 to +231 relative to the start of *rplJ* transcription. The *rplLP'-'lacZ* translational fusion contains nucleotides -382 to +170 relative to the start of *rplJ* transcription. The *rplJL* leader mutations 72-∇UUGAGGUGUA-73, 117-∇UUUGCCUC-118 and G131C:G132C are shown in Fig. 1. All fusions were integrated into the *amyE* locus of the *B. subtilis* chromosome.

[b] Plasmid pVector is the empty vector used to generate plasmids pL10-L12, pLeader and pLeader + L10-L12. Plasmid pL10-L12 contains the *rplJL* coding region under control of an IPTG-inducible promoter. Plasmid pLeader contains the *rplJL* leader (-2 to +200 relative to the start of transcription) under control of the same IPTG-inducible promoter. Plasmid pLeader + L10-L12 contains both of these inserts.

Reference

1. Yakhnin,H., Zhang,H., Yakhnin,A.V. and Babitzke,P. (2004) The *trp* RNA-binding attenuation protein of *Bacillus subtilis* regulates translation of the tryptophan transport gene *trpP* (*yhaG*) by blocking ribosome binding. *J. Bacteriol.* **186**, 278-286.

```
                        ****************         *** *      ******  **  * ******************
B. subtilis             GGAGGCTTTTTATATGGAATCCG--TCG-TCTCAGTCGTGATCACCT-AACGGTATAAGTGTTACACAA
B. atrophaeus           GGAGGCTTTTTATATGTCATTCG--TCGTTCAGAGTCGTGATCACCT-AACGGTATAAGTGTTACACAA
B. amyloliquefaciens    GGAGGCTTTTTATATG-AAATCG--TCG-TCACAGTCGTGATCACCT-AGCGGTATAAGTGTTACACAA
B. licheniformis        GGAGGCTTTTTATATGGAACATG--TCGTTAAAGAACGTGATCACCG-AACGGTATAAGTGTTACACAG
B. pumilus              GGAGGCTTTTTATATGGAGAACCGTTCG-TTAGGAACGTGATGGCCTGATCGGTATAAGTGTTACACAA
                          SD

B. subtilis             MESVVSVVIT
B. atrophaeus           MSFVVQSRDHLTV
B. amyloliquefaciens    MKSSSQS
B. licheniformis        MEHVVKERDHRTV
B. pumilus              MENRSLGT
```

B. kaustophilus

```
             M  S  R  R  Y  K  W  Y  S  L  I  F  L  T  G  G  A  G  R  V  E  R  D
GGAGGTTTTTTATTGAGCAGACGGTATAAGTGGTATTCTCTCATTTTCCTTACAGGAGGTGCAGGACGTGTCGAGCGCGATTGAA...rplJ
  SD                                                                  M  S  S  A  I  E ...L10
```

B. anthracis

```
            M  H  F  R  Y  I  F  Y  I  I  Y  R  R  C  N  N  M ...L10
GGAGGTTTTTAGTGCACTTTCGGTACATCTTCTATATAATCTACAGGAGGTGTAATAACATG...rplJ
  SD
```

Figure S1. *rplJL* leader peptide coding region from *Bacillus* spp. The Shine-Dalgarno sequence (SD), translation initiation and translation stop codons are in bold type. Asterisks (*) represent *rplJL* leader sequences that are conserved in the first five Bacilli. The leader peptide sequences are also shown. The leader peptide coding regions for *B. kaustophilus* and *B. anthracis* have distinct arrangements compared to the other five species.
